# Adenoid cystic carcinoma in palate and maxillary sinus

**DOI:** 10.1016/S1808-8694(15)31008-9

**Published:** 2015-10-19

**Authors:** Vanessa de Fátima Bernardes, Sérgio Vitorino Cardoso, Ricardo Alves Mesquita, Maria Auxiliadora Vieira do Carmo, Maria Cássia Ferreira de Aguiar

**Affiliations:** a DDS, MS in Oral Pathology; b DDS, MS and PhD in Pathology, Professor of Dental Sciences - State University of Montes Claros; c DDS, MS and PhD in Oral Pathology, Assistant Professor - Department of Clinics, Pathology and Surgery in Dental Sciences - Dentistry School - Federal University of Minas Gerais; d DDS, MS and PhD in Oral Pathology, Assistant Professor - Department of Clinics, Pathology and Surgery in Dental Sciences - Dentistry School - Federal University of Minas Gerais; e DDS, MS and PhD in Oral Pathology, Assistant Professor - Department of Clinics, Pathology and Surgery in Dental Sciences - Dentistry School - Federal University of Minas Gerais

**Keywords:** adenoid cystic carcinoma, prognostic factors, salivary gland tumors

## INTRODUCTION

One percent of head and neck malignant neoplasms and 10% of salivary gland neoplasms are adenoid cystic carcinomas (ACC). This tumor frequently occurs in the fifth decade of life, usually affecting women^1^.

The tumor grows slowly, but neural invasion, distance metastases and multiple recurrences are common. This behavior means that ACC patients have a limited prognosis, even after radical surgery and radiotherapy^2^.

We report a case of extensive ACC on the palate, involving the maxillary sinus and the orbit floor. Based of the case report, we discuss the prognosis and the importance of an early diagnosis of ACC.

## CASE PRESENTATION

**Figure 1:**
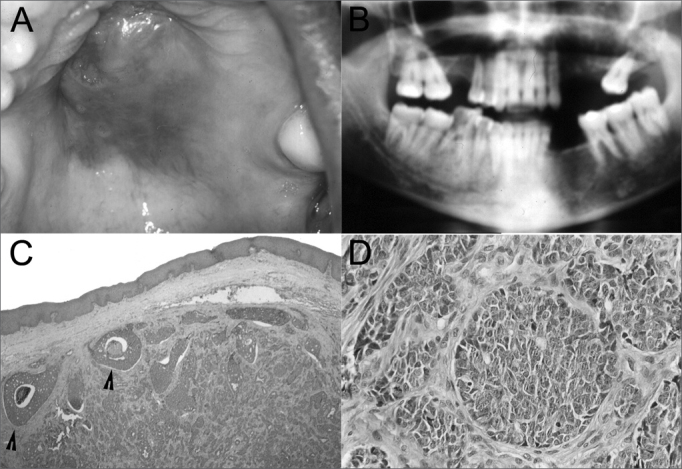
Solid Adenoid Cystic Carcinoma - Figures: **A**. Clinical findings of palate ACC. **B**. Radiology of the lesions. Note the destruction of maxillary walls and extension into the floor of the orbit. **C**, **D**. Microscopy of the lesion - Arrows - Solid lobules with central necrosis.

A female 34-year-old patient presented a lesion on the palate and reported headaches and odynophagia during the past month. The physical exam disclosed a tumor in the mouth with a central ulcer, located on the hard and soft palate, measuring approximately 6cm (Figure 1A). A panoramic radiograph showed bone lysis in teeth 25 to 28, maxillary sinus involvement and extension into the floor of the orbit. (Figure 1B).

A biopsy was made and the specimen was sent to the Pathology Unit. Histopathology revealed a malignant gland epithelial neoplasm characterized by basaloid cell proliferation arranged in nests and solid trabecules. Some nests had central necrosis (Figure 1C). Nuclear pleomorphism, evident nucleoli and mitosis were seen (Figure 1D). The diagnosis was the solid variant of adenoid cystic carcinoma. The patient was referred to the Head & Neck Surgery Unit. Radiotherapy was to be followed by surgical removal of the tumor. The patient, however, died before treatment, due to the tumor.

## DISCUSSION

Many factors should be taken into account in the prognosis of ACC, including the histological and clinical stages of the disease. Greater aggressiveness has been related to the solid variant^3^. Tumor involving the nose, the paranasal sinuses and the maxilla have a worse prognosis^4^. Positive margins and neural infiltration suggest recurrence and negatively influence survival^4^. Limited tumor extension and complete surgical removal may cure the disease, which underlines the need for an early diagnosis^5^.

Neoplastic cell neurotropism causes pain^1^. This was a significant complaint of this patient, together with headaches and odynophagia.

Spiro et al.^6^ observed that patients with ACC involving the maxillary sinus had more advanced disease due to bone and tissue invasion. Our patient also had these findings, which reduced her life expectancy.

## FINAL COMMENTS

The solid variant of ACC is the most aggressive form of this neoplasm. Successful treatment and patient survival are related to the histological stage, tumor location, size and the early diagnosis of the lesion. In this case the findings suggested an unfavorable prognosis for the patient.
